# Awareness of and Attitudes towards Heat Waves within the Context of Climate Change among a Cohort of Residents in Adelaide, Australia

**DOI:** 10.3390/ijerph10010001

**Published:** 2012-12-20

**Authors:** Derick A. Akompab, Peng Bi, Susan Williams, Janet Grant, Iain A. Walker, Martha Augoustinos

**Affiliations:** 1 Discipline of Public Health, School of Population Health, The University of Adelaide, Adelaide 5005, Australia; E-Mails: derick.akompab@adelaide.edu.au (D.A.A.); susan.williams@adelaide.edu.au (S.W.); 2 Population Research & Outcome Studies, The University of Adelaide, Adelaide 5005, Australia; E-Mail: janet.grant@adelaide.edu.au; 3 Climate Adaptation Flagship, Commonwealth Scientific and Industrial Research Organisation, CSIRO, Perth 6151, Australia; E-Mail: iain.a.walker@csiro.au; 4 School of Psychology, The University of Adelaide, Adelaide 5005, Australia; E-Mail: martha.augoustinos@adelaide.edu.au

**Keywords:** climate change, heat waves, human health, attitudes, survey, Australia

## Abstract

Heat waves are a public health concern in Australia and unprecedented heat waves have been recorded in Adelaide over recent years. The aim of this study was to examine the perception and attitudes towards heat waves in the context of climate change among a group of residents in Adelaide, an Australian city with a temperate climate. A cross-sectional study was conducted in the summer of 2012 among a sample of 267 residents. The results of the survey found that television (89.9%), radio (71.2%), newspapers (45.3%) were the main sources from which respondents received information about heat waves. The majority of the respondents (73.0%) followed news about heat waves very or somewhat closely. About 26.6% of the respondents were extremely or very concerned about the effects of heat waves on them personally. The main issues that were of personal concern for respondents during a heat wave were their personal comfort (60.7%), their garden (48.7%), and sleeping well (47.6%). Overall, respondents were more concerned about the impacts of heat waves to the society than on themselves. There was a significant association between gender (χ² = 21.2, df = 3, *p* = 0.000), gross annual household income (*p* = 0.03) and concern for the societal effects of heat waves. Less than half (43.2%) of the respondents believed that heat waves will extremely or very likely increase in Adelaide according to climate projections. Nearly half (49.3%) believed that the effects of heat waves were already being felt in Adelaide. These findings may inform the reframing and communication strategies for heat waves in Adelaide in the context of climate change.

## 1. Introduction

Climate change has been the subject of much discussion over the past decades and it poses a great challenge to human health and societal well-being [1,2]. There is a growing body of scientific research suggesting that extreme weather events (e.g., heat waves) will become more frequent as a result of climate change [3,4]. For example, in the United States about fourteen record-breaking weather and climate-related disasters which caused economic losses and loss of human life were reported in 2011. Heat waves were among these weather-related disasters and in March 2011, about 15,292 warm temperature records were broken across the United States [5]. Heat waves are a major public health concern in many temperate regions and episodes recorded around the world have shed further light on the health, social, environmental and economic consequences associated with heat waves [6,7,8].

Across Australia, a number of cities, including Adelaide, usually experience heat waves during the months of summer. The most recent and significant heat wave was recorded between late January and early February 2009 which affected most parts of south-eastern Australia [9], and is still fresh in the minds of many. The early 2009 heat wave (the South Australian Bureau of Meteorology—BoM—defines a heat wave as a period of maximum temperatures of 35 °C or over for a period of five or more consecutive days or three or more consecutive days of temperatures of 40 °C or above) resulted in an estimated 500 heat-related deaths in South Australia and Victoria in addition to the social, economic and environmental consequences [10,11]. Apart from the unprecedented early 2009 heat wave, Adelaide experienced other major heat waves in 2008 and 2010. The 2008 and 2009 heat waves were unique in terms of their duration (15 days and 13 days respectively), and the 2009 heat wave was also remarkable in its intensity as there were six consecutive days with temperatures over 40 °C and one day with a maximum temperature of 45.7 °C [12,13]. In the 2010 heat wave, Adelaide recorded five consecutive days in excess of 35 °C with four of these days exceeding 40 °C [14]. Monthly maximum temperatures for Adelaide during summer and early autumn (December to March) normally range from 27.0 °C–29.4 °C [13]. Climate models project that heat waves will increase in frequency and severity in Adelaide as a consequence of climate change [15].

Authorities in Adelaide and in other regions of the world are aware of the risks posed by heat waves. Epidemiological studies have shown that certain groups within the population are more vulnerable to heat waves because of a number of underlying physiological and contextual factors [16,17,18]. Climate-related impacts also cause emotional and psychological distress to many people, especially to those who are vulnerable [19,20]. By the same token, studies have reported that people are concerned about the impacts of climate change [21]. However, studies also show that individuals distinguish between the effects of climate change on their personal lives and on society. While some people have expressed more concerned for personal issues such as their health, finance, personal comforts and safety [22,23], others may be more concerned about societal impacts of climate change with the belief that the risks associated with these impacts are greater for people who are spatially or temporally distant [24]. It is also important to bear in mind that the link between heat waves and global climate change has been the subject of discussion among the public since the science of climate change is complex and still contested [25]. Furthermore, people’s attitudes and beliefs about climate-related risks such as heat wave may be shaped by their pre-existing knowledge and information they receive from many sources and their every day interaction within the society [26].

There has been growing interest in understanding public views about environmental issues. For example, a number of studies have examined public perception and attitudes towards climate change [27,28,29,30] and most of these studies have found that public attitudes towards climate change have varied over the years. Other studies have examined perceptions about heat waves [31,32,33,34,35] and most of these studies found that some participants didn’t belief that they were vulnerable to heat waves. Although heat waves are common in Australia, there are limited studies that have specifically examined public attitudes towards heat waves in relation to climate change.

The aim of this study was therefore to examine public attitudes towards heat waves in the context of climate change in Adelaide. Heat waves have been common recently in Adelaide, thus making it an ideal city for such a study to be conducted. Understanding public attitudes may be important as it would inform public education and communication strategies for heat waves under a changing climate. Furthermore, having an understanding of public attitudes may be important since any success to address potential consequences associated with heat waves may depend on public views about the phenomenon. This study draws insights from previous surveys on climate change and applies these to heat waves. The findings are part of a larger research study conducted in Adelaide.

## 2. Methods

A questionnaire was developed after a review of the literature on heat waves and climate change. Some questions and response options were informed by previous surveys on public attitudes towards climate change [27,28,29,30]. All the questions were however adapted to the context of heat waves, with some questions having both closed and open-ended responses. The draft questionnaire was validated by experts and piloted among a selection of 20 residents in Adelaide. Major revisions were made to the draft questionnaire to facilitate ease and understanding before it was finalised.

The study population from which the sample was drawn was the North West Adelaide Health Study cohort, a representative group of adults residing in the north and western region of Adelaide. This is a pre-existing group of individuals who have been participating in health research [36]. Ethics approval was obtained from The University of Adelaide Human Research Ethics Committee (No. H-061-2011) and The Queen Elizabeth Hospital Human Research Ethics Committee (No. 2011136); since the latter oversees any research study related to the cohort.

With support of the chief investigators of this cohort, and as part of a follow-up survey, study participants were asked if they would be willing to be contacted at a later date to take part in a questionnaire study on heat waves. Of the 1,185 approached, 818 expressed interest to participate in the heat waves study. Among those who expressed interest, 490 participants were selected. The selection criteria used was being aged between 30–69 years and having the literacy skills needed to self-complete a questionnaire. Questionnaires were mailed out to selected participants in late January 2012, together with the study information sheet and participants were requested to return their completed questionnaire using a supplied replied-paid envelope.

The questionnaires were mailed during the summer, following weeks of hot weather in Adelaide where temperatures were above 30 °C [37] which ensured that the hot weather was salient to respondents at the time. Completing the questionnaire was construed as providing consent to take part in the study. Of the 490 questionnaires which were mailed out, 272 were returned giving a response rate of 55.5%. No follow-up or reminder calls were made to the respondents. Due to missing data, five questionnaires were eliminated leaving 267 for final analysis.

Data were entered into an Access database and “imported” into Stata version 12 (Stata Corp, College Station, TX, USA) for analysis where descriptive and bivariate analysis was performed. In bivariate analysis, Chi square or Fisher’s exact tests (expected cell frequencies less than or equal to five) were used to test for the inter-relationships among variables with a *p*-value < 0.05 considered to be statistically significant. No statistical weighting of the data was performed. Some categorical demographic variables (e.g., marital status, level of education, employment status) were dichotomized because of sample size constraints.

## 3. Results

### 3.1. Respondents’ Demographic Characteristics

Table 1 shows selected demographic characteristics of respondents. The mean age of the respondents was 51 years, with a majority of them (61.4%) in the age group 50–69 years. More than half of the respondents (55.8%) were female. A majority of the respondents (89.1%) were married. In terms of level of education, more than half of the respondents (61.4%) did some training after high school or further education (bachelor or postgraduate degree).

**Table 1:** Selected demographic characteristics of respondents.

Variable	Number	Percent (%)
**Age group (years) (n = 267)**		
30–49	103	38.6
50–69 (Mean = 51 years)	164	61.4
**Gender(n = 267)**		
Male	118	44.2
Female	149	55.8
**Marital status (n = 267)**		
Never married	29	10.9
Married ^1^	238	89.1
**Level of Education (n = 267)**		
Educated at or below high school level	103	38.6
Educated above high school level ^2^	164	61.4
**Employment Status (n** **= 267)**		
Not employed ^3^	43	16.1
Employed	224	83.9
**Gross annual household income (n** **= 254)**		
Less than $40,000	58	22.8
Between $40,000 and $59,999	53	20.9
$60,000 or more	143	56.3

^1^ Married (individuals married at some point in life and include currently married, separated and widowed); ^2^ Referred to those who did additional training after high school (e.g., TAFE courses, bachelor or postgraduate degree); ^3^ Not employed referred to unemployed, retired and home duties.

### 3.2. Awareness about Heat Waves

**Figure 1:**
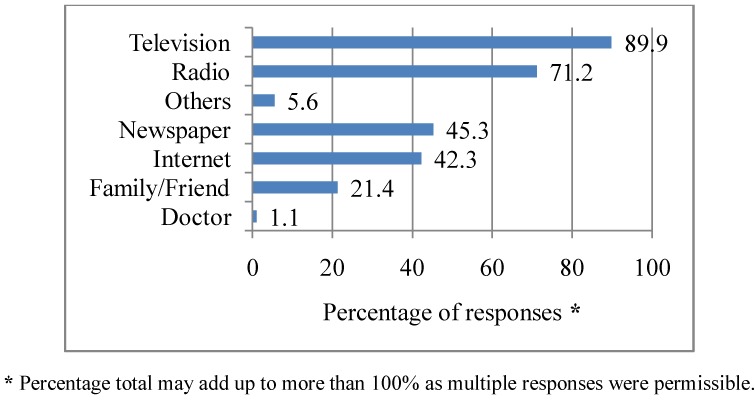
Sources of information about heat waves. Q1: Where do you normally get information about heat waves? (multiple responses).

Respondents were asked where they usually get information about heat waves. Television was the most commonly cited source of information (89.9%), followed by the radio (71.2%), newspapers (45.3%), the internet (42.3%). The other sources (5.6%) from which respondents indicated they obtained news about heat waves included: the Australian Bureau of Meteorology, mobile phone web applications, newsletters, leaflets and brochures. Further details are illustrated in Figure 1.

### 3.3. Degree to Which Information about Heat Waves is Followed

**Figure 2:**
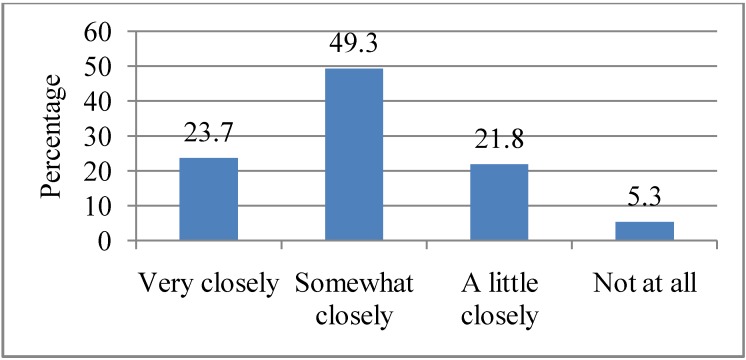
Degree to which respondents follow news about heat waves. Q2. How closely do you follow news about heat waves from these sources?

Respondents were asked how closely they followed information from these sources during a heat wave. Nearly half (49.3%) indicated that they followed information “Somewhat closely”, 23.7% followed information “Very closely”, 21.8% followed information “A little closely” and 5.3% indicated that they do not follow information about heat waves at all (see Figure 2). There was a marginally significant association between gender and the closeness to which respondents followed news about heat waves (*p* = 0.05), with women (62.0%) more likely to follow “Very closely” news about heat waves than men.

### 3.4. Level of Information about Heat Waves

**Figure 3:**
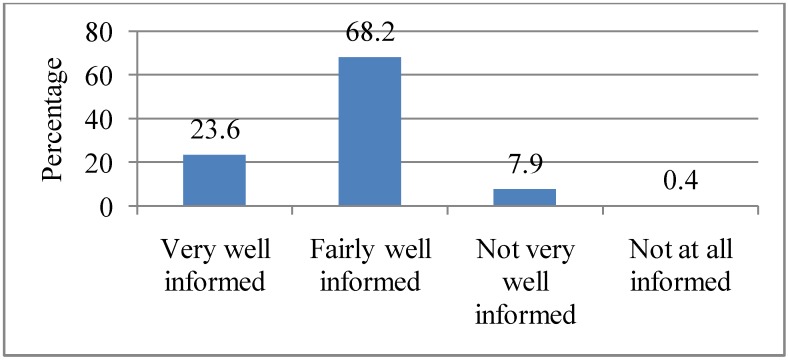
Extent to which respondents feel informed about heat waves and its consequences. Q3. How well informed do you think you are about heat waves and its consequences?

Since most of the respondents indicated getting information from at least one of the above information sources, they were asked about how well informed they were about heat waves. More than half (68.2%) cited being “Fairly well informed”; followed by those who were “Very well informed” (23.6%). The rest of the respondents were either “Not very well informed” (7.9%) or “Not at all informed” (0.4%), as illustrated in Figure 3. There was a statistically significant association between the closeness to which news about heat waves was followed and the level of information about heat waves (*p* = 0.000). Those who were “Fairly well informed” about heat waves (57.1%) were more likely to follow “Very closely” news about heat waves.

### 3.5. Personal Concern about the Effects of Heat Waves

**Figure 4:**
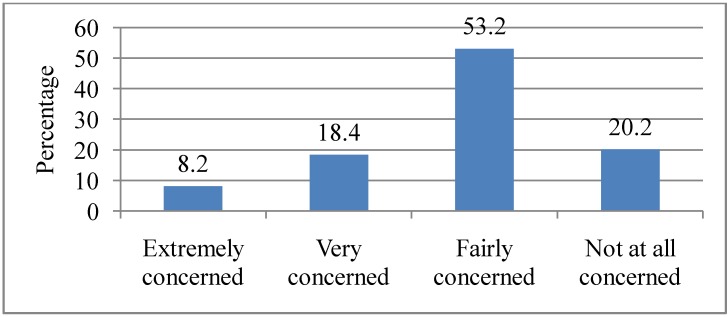
Extent to which respondents are personally concerned about the effects of heat waves. Q4. How concerned are you about the effects of heat waves on you (personally)?

Respondents were then asked how they were personally concerned about the effects of heat waves. A majority of them (53.2%) cited being “Fairly concerned”, followed by those who were “Not at all concerned” (20.2%). While 18.4% of the respondents were “Very concerned”, only 8.2% were “Extremely concerned” about the effects of heat waves on them personally (see Figure 4). There was no statistically significant association between the socio-demographic variables and personal concern for heat waves.

### 3.6. Issues That Are of Concern to Respondents during a Heat Wave

**Figure 5:**
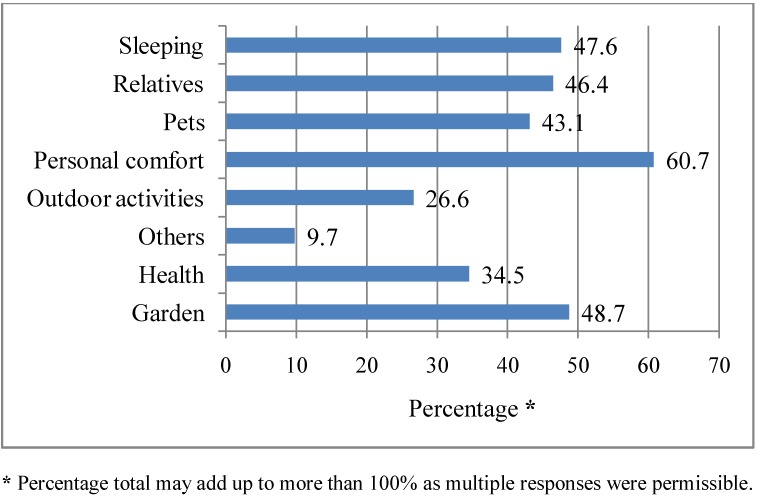
Respondents concerns during a heat wave. Q5. What are you concerned about during a heat wave? (multiple responses).

Those respondents who were either “Extremely, Very or Fairly concerned” (*i.e.*, approximately 213 or 79.8% of the total respondents) about the effects heat waves may have on them personally were asked what they were particularly concerned about during a heat wave. Respondents indicated being concerned about their personal comfort (60.7%), garden (48.7%), sleeping well (47.6%), relatives (46.4%), pets (43.1%), health (34.5%) and outdoor activities (26.6%). The other concern (9.7%) cited included the cost of running an air-conditioner as well as the possibility of developing sun burn during a heat wave (see Figure 5). There was a statistically significant association between gender and concern for their relatives (χ² = 4.73, df = 1, *p* = 0.03) during a heat wave, with women (62.9%) more likely to be concerned about their relatives. There was also a marginally significant association between gender and concern over the safety of pets (χ² = 3.79, df = 1, *p* = 0.05) during a heat wave, with women (62.6%) more likely to be concerned about their pets than men.

### 3.7. Extent of Concern about Societal Effects of Heat Waves in Adelaide

**Figure 6:**
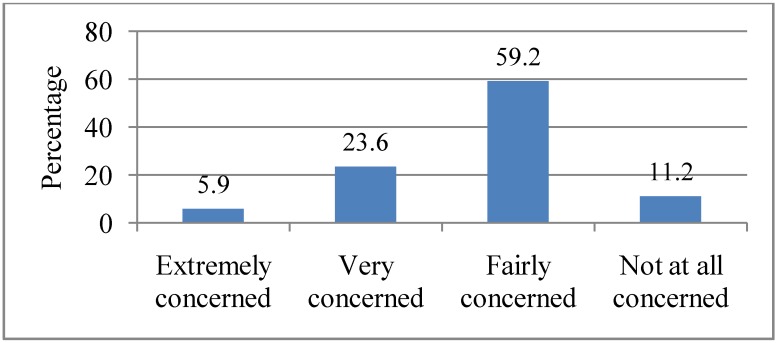
Extent of respondents’ societal concern on the impacts of heat waves. Q6. How concerned are you about the effects of heat waves to society?

All respondents were asked how concerned they were about the effects of heat waves to the society at large. Only 5.9% indicated being “Extremely concerned” while more than half (59.2%) indicated being “Fairly concerned”. The rest were either “Very concerned” (23.6%) or “Not at all concerned” (11.2%), as illustrated in Figure 6. There was a statistically significant association between gender (χ² = 21.2, df = 3, *p* = 0.000), gross annual household income (*p* = 0.03) and concern for societal effects of heat waves. Women (80.9%) were more likely to be “Very concerned” about the societal effects of heat waves than men. On the other hand, men (52.5%) were more likely to be “Fairly concerned” about the societal effects than women. Respondents with a gross annual household income of ≥$60,000 (60.93%) were more likely to be “Fairly concerned” about the societal effects of heat waves. There was also a statistically significant association between level of information about heat waves and concern for the societal effects of heat waves (*p* = 0.005). Those who were “Very well informed” (25.4%) were more likely to be “Very concerned” about the societal effects of heat waves, while those who were “Fairly well informed” (63.3%) were less likely to be concerned about the effects of heat waves to society.

### 3.8. Effects of Heat Waves on Respondents’ Wellbeing

**Figure 7:**
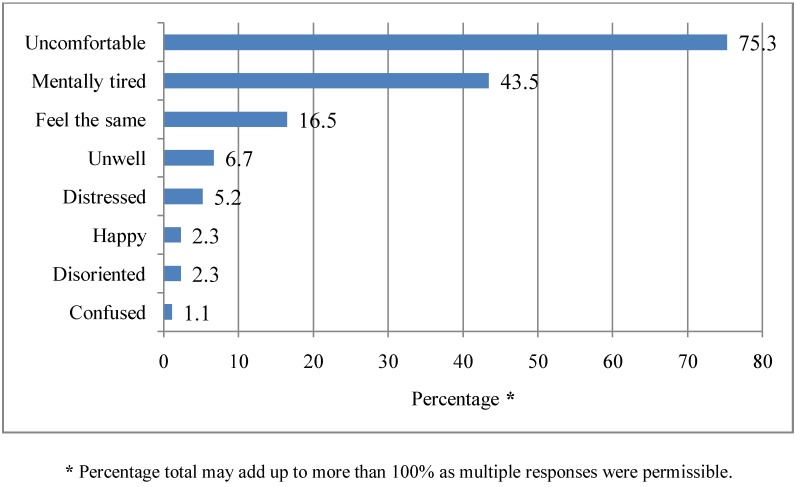
Respondents’ wellbeing during a heat wave. Q7. How do you feel during a heat wave? (multiple responses).

Respondents were asked about how they feel during periods of heat waves in Adelaide. The most cited responses were feeling “uncomfortable” (75.3%), followed by “mentally tired” (43.5%), “feeling the same as usual” (16.5%) and feeling “unwell” (6.7%). The least common cited response was “feeling disoriented” (2.3%), happy (2.3%) and “feeling confused” (1.1%). These responses are illustrated in Figure 7. There was a statistically significant association between gender (χ² = 14.4, df = 1, *p* = 0.000), gross annual household income (χ² = 10.38, df = 1, *p* = 0.006) and being “mentally tired” during a heat wave, with women (68.9%) and those with a gross annual household income of ≥$60,000 (45.5%) more likely to be “mentally tired”. There was a statistically significant association between age (χ² = 3.9, df = 1, *p* = 0.04), gender (χ² = 5.9, df = 1, *p* = 0.01) and feeling “unwell” during a heat wave, with those aged over 49 years (83.3%) and women (83.2%) more likely to feel “unwell”.

### 3.9. Attitudes towards Scientific Projections of Increasing Heat Waves in Relation to Climate Change

**Figure 8:**
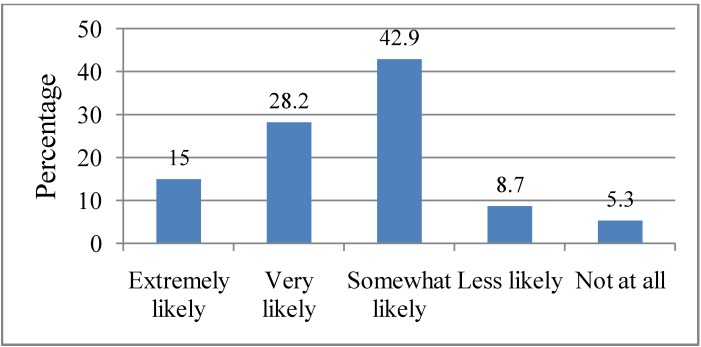
Respondents’ attitudes towards scientific projections about heat waves. Q8. How likely do you think heat waves in Adelaide will increase in the future as some scientists have projected?

Scientific projections hold that heat waves are likely to increase in frequency, intensity and duration as a result of global climate change. Respondents were asked how likely they believed heat waves will increase in the future according to these projections. Fifteen percent (15.0%) indicated that it would be “Extremely likely” for heat waves to increase in future according to scientific projections. The other respondents indicated that it will be “Very likely” (28.2%), “Somewhat likely” (42.9%), “Less likely” (8.7%) and “Not at all” (5.3%). These responses are illustrated in Figure 8. There was a statistically significant association between marital status (*p* = 0.04) and belief in the likelihood of increasing heat waves in Adelaide, with those married (80.0%) more likely to believe that it will be “Extremely likely” that heat waves will increase in future according to scientific projections. There was also a marginally significant association between employment status (*p* = 0.05) and belief in the likelihood of increasing heat waves in Adelaide, with those employed (67.5%) more likely to believe that it will be “Very likely” that heat waves will increase in future. There was no significant association between level of education and the belief in the likelihood of increasing heat waves in Adelaide.

### 3.10. Attitudes towards Potential Consequences of Heat Waves

**Figure 9:**
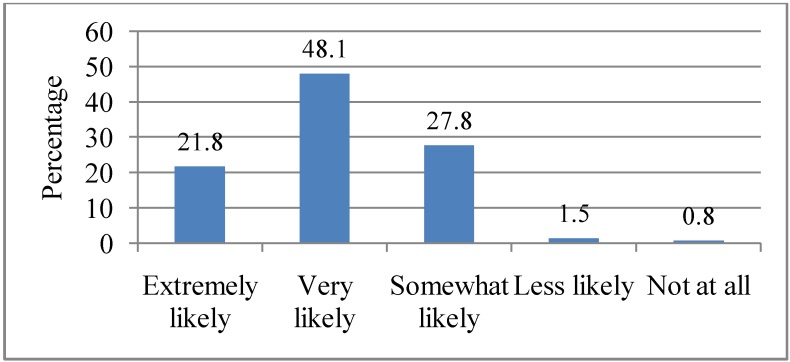
Respondents’ attitudes towards potential consequences of heat waves. Q9. If heat waves were to increase in future, how likely do you think this will have consequences in Adelaide?

Heat waves in Adelaide have been associated with morbidity and mortality as well as social, economic and environmental consequences. Respondents were asked how likely they believed that heat waves would have consequences if heat waves were to increase in future. Nearly half of them (48.1%) cited that the consequences would be “Very likely”, 27.8% indicated that the consequences would be “Somewhat likely”, followed by those who noted that it would be “Extremely likely” (21.8%). Only a minority indicated that it would be “Less likely”(1.5%), while 0.8% cited “Not at all”, as illustrated in Figure 9.

There was a statistically significant association between marital status (*p* = 0.03), employment status (*p* = 0.001) and the belief that future heat waves will have severe consequences. Respondents who were married (93.7%) and employed (84.4%) were more likely to believe that it will be “Very likely” that future heat waves will have severe consequences in Adelaide.

### 3.11. Immediacy of the Effects of Heat Waves in Adelaide

**Figure 10:**
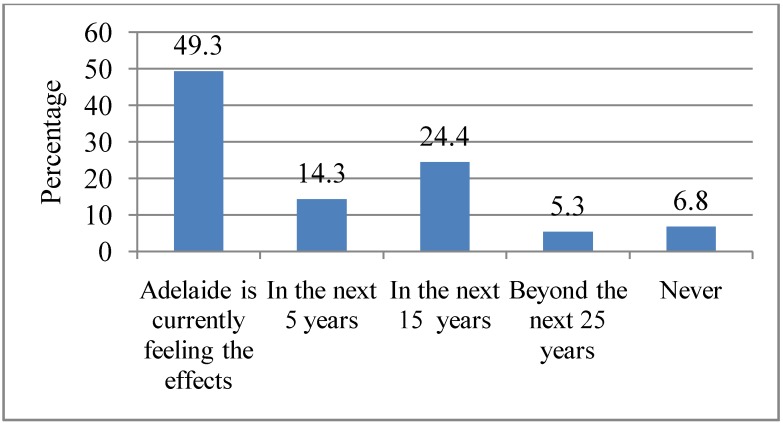
Respondents’ attitudes towards immediacy of the impacts of heat waves. Q10. When, if at all, do you think Adelaide will start feeling the social and health effects of heat waves?

Respondents were asked to indicate when they believed the social and health effects of heat waves would be felt in Adelaide. Almost half of them (49.3%) indicated that heat waves were already causing social and health effects in Adelaide; 14.3% of the respondents indicated that the effects will be felt in 5 years time, 24.4% indicated that the effects will be felt in 15 years time, while 5.3% indicated that the effects will be felt beyond the next 25 years and 6.8% indicated that Adelaide will never suffer from any social or health effects associated with heat waves. These responses are shown in Figure 10. There was a marginally significant association between gender (*p* = 0.05) and those who believed that Adelaide was already feeling the effects of heat waves, with women (56.0%) more likely to believe that “Adelaide was already feeling the effects” of heat waves.

## 4. Discussion

The objectives of this study were to examine the perception and attitudes towards heat waves within the context of climate change. Overall, respondents reported that they were well informed about heat waves and concerned about the effects of heat waves. However, attitudes varied over some of the issues examined in the survey.

It was found that television (TV), radio, newspaper and internet were the main sources from which respondents obtained information during a heat wave. Similar findings were obtained in a study on heat awareness across four North American cities. However, in that study, the internet was relatively unused by respondents with only 3.0% reported using the internet as a source for heat advisories [31]. Another study conducted in the United States to assess the source of weather advisories found that TV, radio, and newspaper were the major sources of information [33]. This finding highlights the important role of the media as a source from which the public obtains information about heat waves and this likely has implications for policy. It underscores the need for policy makers and emergency services providers to make appropriate use of the media to disseminate information before, during and after a heat wave.

Less than a quarter of the respondents expressed being “Extremely concerned” (8.2%) and “Very concerned” (18.4%) about the threat of heat waves on them personally. Among those who were personally concerned, they indicated concern for their personal comfort, their garden, sleeping, relatives and their pets. Although heat waves may lead to heat-related illness and mortality, less than half (34.5%) of the respondents indicated health as a concern during a heat wave. This perhaps indicates that respondents did not consider heat waves as an immediate threat to their health. This study also found that the majority of respondents did not have a very high level of concern about the effects of heat waves on society. However, more than half of them (59.2%) indicated that they were “Fairly concerned” about the effects of heat waves on society. Overall, there appeared to be a higher level of concern for the effects of heat waves on society than on personal issues (*i.e.*, 20.2% *vs*. 11.2% “Not at all concerned” respectively for personal and societal effects of heat waves). Heat waves are an environmental threat and studies have generally found that women are more likely to be very concern than men about environmental issues which pose a risk for vulnerable groups [38,39,40]. We found that respondents with a gross annual household income of ≥$60,000 were more likely to be “Fairly concerned” about the societal effects of heat waves. However, some studies have found that income was negatively related to concern for environmental problems [39,40]. The study also found that women were more likely to be concerned about the safety of their relatives and pets, which could be explained by their family role and domestic responsibilities to provide care for children, elderly relatives and take care of household belongings.

The results of this study showed that heat waves have an impact on the wellbeing of residents in Adelaide. Some respondents cited being mentally tired and/or being distressed during a heat wave; which probably indicates that heat waves may likely affect the emotional wellbeing of certain individuals. For example, some individuals might be distressed when they watch apocalyptic images of bushfires associated with heat waves or be distressed when their garden dries-off or their pet dies as a result of a heat wave. The most common response cited was feeling uncomfortable during a heat wave. When people are uncomfortable during a heat wave, it may affect their mood; they may develop negative feelings, anxiety and worry because of the heat wave. Since heat waves are an environmental stressor, studies have found that women tend to be more emotionally or psychologically affected during a stressful event [41,42]. For example, there are studies that have shown that women are more vulnerable to the effects of heat waves [43,44] although contrasting findings have also been reported [18]. It has been suggested that gender differences in emotional or psychological distress may be explained by differences in the way women and men perceive and express distressful events [45].

A majority of the respondents indicated that there was a likelihood that heat waves will increase in future as some scientists have projected, although there was a variation in terms of the degree of likelihood. Only a minority of respondents indicated that the chances of heat waves increasing would either be less likely (8.7%) or “Not at all” (5.3%). It may be possible that respondents’ responses to this question may have been influenced by either their past experience of heat waves or based on their scientific views about climate change as a whole.

Most of the respondents indicated that it would be quite likely that if heat waves increase in Adelaide, it would have severe health consequences. Only a minority (1.5%) stated that it would be less likely or not at all likely (0.8%) for any consequences to occur. A possible explanation could be that respondents had perhaps experienced or read reports of health impacts during recent heat waves in Adelaide. A qualitative study was conducted to explore participants’ mental model about the consequences of heat waves in Adelaide. Participants indicated that heat waves were associated with health, social, environmental, psychological and emotional consequences [46]. An interesting finding from this study was about the immediacy of the effects of heat waves. Almost half (49.3%) of the respondents indicated that the effects of heat wave were currently being felt in Adelaide. Again, this probably reflects their experiences during recent heat waves. For example, the early 2009 heat wave resulted in heat-related deaths, hospitalisation, closure of schools, disruption of public transport system and power black-outs [12,47,48]. As a result, some of the respondents may have either been affected or known someone who was affected during the heat wave. These experiences may have motivated them to believe that heat waves are already having an effect in Adelaide.

The present findings may be limited by this study’s cross-sectional design and the results cannot be generalised to the entire Adelaide population since those who participated reside in a specific region. Despite this limitation, these findings are useful as they provide information about the attitudes that exist within a community about heat waves and identify the major concerns and psychological issues that affect residents during a heat wave. Such information may be useful for authorities during planning and responding to heat waves. Future studies should be conducted to examine public attitudes towards heat waves in relation to climate change in other cities or regions of the world that usually experience heat waves. This is because attitudes may vary according to climate and geographic locations. Lastly, repeated studies should be conducted to examine any change in attitudes over time.

## 5. Conclusions

The results of this survey suggest that people are well informed about heat waves and have experienced past episodes of heat waves in the city. The media was the main source from which respondents obtained information about heat waves. This finding indicates the important role that the media plays in disseminating information to the public. Some individuals are concerned over certain issues during a heat wave, with some concerned about the societal impacts of heat waves. Heat wave is a threat associated with certain emotional and psychological distress among certain groups in the community. The public’s attitude towards certain issues related to heat waves may be influenced by their beliefs about global climate change.

## References

[1] Myers S., Patz J. (2009). Emerging threats to human health from global environmental change. Annu. Rev. Environ. Resour..

[2] Costello A., Abbas M., Allen A., Ball S., Bellamy R., Friel S., Groce N. (2009). Managing the health effects of climate change. Lancet.

[3] Solomon S., Qin D., Manning M., Chen Z., Marquis M., Averyt K.B., Tignor M., Miller H.L., IPCC (2007). Summary for Policy Makers. Climate Change 2007: The Physical Science Basis. Contribution of Working Group I to the Fourth Assessment Report of the Intergovernmental Panel on Climate Change.

[4] Easterling D., Evans J., Groisman P., Karl T., Kunkel K., Ambenje P. (2000). Observed variability and trends in extreme climate events. Bull. Am. Meteorol Soc..

[5] Leiserowitz A., Maibach E., Roser-Renouf C., Hmielowski J. (2012). Extreme Weather, Climate & Preparedness in the American Mind.

[6] Poumadere M., Mays C., le Mer S., Blong R. (2005). The 2003 heat wave in France: Dangerous climate change here and now. Risk Anal..

[7] 7. Dole R., Hoerling M., Perlwitz J., Eischeid J., Pegion P., Zhang T., Quan X.W., Xu T., Murray D. (2011). Was there a basis for anticipating the 2010 Russian heat wave?. Geophys Res. Lett..

[8] Hegerl G.C., Zwiers F.W., Braconnot P., Gillett N.P., Luo Y., Orsini J.A.M., Nicholls N., Penner J.E., Stott P.A., Solomon S., Qin D., Manning M., Chen Z., Marquis M., Averyt K.B., Tignor M., Miller H.L. (2007). Understanding and Attributing Climate Change. Climate Change 2007: The Physical Science Basis. Contribution of Working Group I to the Fourth Assessment Report of the Intergovernmental Panel on Climate Change.

[9] Australian Government: Bureau of Meteorology The Exceptional January-February 2009 Heatwave in South-eastern Australia (2009). www.bom.gov.au/climate/current/statements/scs17c.pdf.

[10] Reeves J., Foelz C., Grace P., Best P., Marcussen T., Mushtaq S., Stone R., Loughnan M., McEvoy D., Ahmed I., Mullett J., Haynes K., Bird D., Coates L., Ling M. (2010). Impacts and Adaptation Response of Infrastructure and Communities to Heatwaves: The Southern Australian Experience of 2009.

[11] Parliament of Victoria 2009 Victorian Bushfires Royal Commission, Final Report, Volume 1. Government Printer for the State of Victoria, Melbourne. www.royalcommission.vic.gov.au/Commission-Reports/Final-Report/Summary/Interactive-Version.

[12] Nitschke M., Tucker G., Hansen A., Williams S., Zhang Y., Bi P. (2011). Impact of two recent extreme heat episodes on morbidity and mortality in Adelaide, South Australia: A case-series analysis. Environ. Health.

[13] Australian Government: Bureau of Meteorology Climate Statistics for Australian Locations. Summary Statistics for ADELAIDE. www.bom.gov.au/climate/averages/tables/cw_023090.shtml.

[14] Australian Government: Bureau of Meteorology First Heatwave for Adelaide in 2010. www.bom.gov.au/announcements/media_releases/sa/20100115_First_Heatwave_SA_Jan.shtml.

[15] Suppiah R., Hennessy K., Whetton P., McInnes K., Macadam I., Bathols J., Ricketts J., Page C. (2007). Australian climate change projections derived from simulations performed for the IPCC 4th Assessment Report. Aust Met. Mag.

[16] Michelozzi P., de’Donato F., Bisanti L., Riusso A., Cadum E., Demaria M., D’Ovidio M., Costa G., Perucci C. (2005). The impact of the summer 2003 heat waves on mortality in four Italian cities. Euro Surveill.

[17] Stafoggia M., Forastiere F., Agostini D., Caranci N., de’Donato F., Demaria M., Michelozzi P., Miglio R., Rognoni M., Russo A., Perucci C. (2008). Factors affecting in-hospital heat-related mortality: A multi-city case-crossover analysis. J. Epidemiol. Community Health.

[18] Bell M.L., O’Neill M.S., Ranjit N., Borja-Aburto V.H., Cifuentes L.A., Gouveia N.C. (2008). Vulnerability to heat-related mortality in Latin America: A case-crossover study in Sao Paulo, Brazil, Santiago, Chile and Mexico City, Mexico. Int. J. Epidemiol..

[19] Fritze J., Blashki G., Burke S., Wiseman J. (2008). Hope, despair and transformation: Climate change and the promotion of mental health and well being. Int. J. Ment. Health Syst..

[20] Lorenzoni I., Leiserowitz A., de Franca Doria M., Poortinga W., Pidgeon N. (2006). Cross-national comparisons of image associations with “global warming” and “climate change” among laypeople in the United States of America and Great Britain. J. Risk Res..

[21] Searle K., Kathryn Gow K. (2010). Do concerns about climate change lead to distress?. Int. J. Clim. Change Strat. Manage..

[22] Bickerstaff K., Simmons P., Pidgeon N. (2004). Public Perceptions of Risk, Science and Governance: Main Findings of a Qualitative Study of Five Risk Cases.

[23] Poortinga W., Pidgeon N. (2003). Public Perceptions of Risk, Science and Governance—Main Findings of a British Survey on Five Risk Cases, Technical Report, Centre for Environmental Risk.

[24] Upham P., Whitmarsh L., Poortinga W., Purdam K., Darnton A., McLachlan C., Devine-Wright P. (2009). Public Attitudes to Environmental Change: A Selective Review of Theory and Practice.

[25] Ungar S. (2000). Knowledge, ignorance and the popular culture: Climate change *versus* the ozone hole. Public Underst. Sci..

[26] Carvalho A. (2007). Ideological cultures and media discourses on scientific knowledge: Re-reading news on climate change. Public Underst. Sci..

[27] Akerlof K., DeBono R., Berry P., Leiserowitz A., Roser-Renouf C., Clarke K.L., Rogaeva A., Nisbet M.C., Weathers M.R., Maibach E.W. (2010). Public perceptions of climate change as a human health risk: Surveys of the United States, Canada and Malta. Int. J. Environ. Res. Public Health.

[28] Leiserowitz A., Smith N., Marlon J. (2010). Americans’ Knowledge of Climate Change.

[29] Reser J., Pidgeon N., Spence A., Bradley G., Glendon A., Ellul M. (2011). Public Risk Perceptions, Understandings, and Responses to Climate Change in Australia and Great Britain: Interim Report.

[30] Leviston Z., Walker I.A. (2011). Baseline Survey of Australian Attitudes to Climate Change: Preliminary Report.

[31] Sheridan S.C. (2007). A survey of public perception and response to heat warnings across four North American cities: An evaluation of municipal effectiveness. Int. J. Biometeorol..

[32] Chowdhury P.D., Haque C.E., Driedger S.M. (2011). Public *versus* expert knowledge and perception of climate change-induced heat wave risk: A modified mental model approach. J. Risk Res..

[33] Semenza J.C., Wilson D.J., Parra J., Bontempo B.D., Hart M., Sailor D.J., George L.A. (2008). Public perception and behavior change in relationship to hot weather and air pollution. Environ. Res..

[34] Hansen A., Bi P., Nitschke M., Pisaniello D., Newbury J., Kitson A. (2011). Perceptions of heat-susceptibility in older persons: Barriers to adaptation. Int. J. Environ. Res. Public Health.

[35] Abrahamson V., Wolf J., Lorenzoni I., Fenn B., Kovats S., Wilkinson P., Adger N.W., Raine R. (2009). Perception of heatwaves risks to health: Interview-based study of older people in London and Norwish, UK. J. Public Health.

[36] Grant J.F., Chittleborough C.R., Taylor A.W., Grande E.D., Wilson D.H., Phillips P.J., Adams R.J., Cheek J., Price K., Gill T., Ruffin R.E. (2006). The North West Adelaide Health Study: detailed methods and baseline segmentation of a cohort for selected chronic diseases. Epidemiol. Perspect. Innov..

[37] Australian Government:Bureau of Meteorology Daily Maximum Temperatures for Adelaide. www.bom.gov.au/climate/data/.

[38] Brody S., Zahran S., Vedlitz A., Grover H. (2008). Examining the relationship between physical vulnerability and public perceptions of global climate change in the United States. Environ. Behav..

[39] O’Connor R., Bord R., Fisher A. (1999). Risk perceptions, general environmental beliefs, and willingness to address climate change. Risk Anal..

[40] McCright A. (2010). The effects of gender on climate change knowledge and concern in the American public. Pop. Environ..

[41] Ensminger M., Celentano D. (1990). Gender differences in the effect of unemployment on psychological distress. Soc. Sci. Med..

[42] Emslie C., Fuhrer R., Hunt K., Macintyre S;. Shipley, Stansfeld S. (2002). Gender differences in mental health: Evidence from three organisations. Soc. Sci. Med..

[43] Donoghue E., Kalelkar M., Boehmer M. (1995). Heat-related mortality-Chicago, July 1995. MMWR.

[44] Vandentorren S., Bretin P., Zeghnoun A., Mandereau-Bruno L., Croisier A., Cochet C., Ribéron J., Siberan I., Declercq B., Ledrans M. (2006). August 2003 heat wave in France: Risk factors for death of elderly people living at home. Eur. J. Public Health.

[45] Drapeau A., Beaulieu-Prévost D., Marchand A., Boyer R., Préville M., Kairouz S. (2010). A life-course and time perspective on the construct validity of psychological distress in women and men. Measurement invariance of the K6 across gender. BMC Med. Res. Method.

[46] Akompab D.A., Bi P., Williams S., Saniotis A., Walker I.A., Augoustinos M. (2012). Climate change, community understanding and emotional responses to the impacts of heat waves in Adelaide, Australia. Int. J. Clim. Change Imp. Responses.

[47] Ham L. (2009). Tracks buckle and so does the rail system. The Age Newspaper.

[48] Owen M., Novak L., Malinauskas R. (2008). Rail commuters stranded in heat. Adelaide Now.

